# Novelty Seeking, Harm Avoidance, and Cerebral Responses to Conflict Anticipation: An Exploratory Study

**DOI:** 10.3389/fnhum.2016.00546

**Published:** 2016-11-03

**Authors:** Jianping Hu, Sien Hu, Julianna R. Maisano, Herta H. Chao, Sheng Zhang, Chiang-Shan R. Li

**Affiliations:** ^1^Laboratory for Behavioral and Regional Finance, Guangdong University of FinanceGuangzhou, China; ^2^Department of Psychiatry, Yale University School of MedicineNew Haven, CT, USA; ^3^Department of Internal Medicine, Yale University School of MedicineNew Haven, CT, USA; ^4^Medical Service, VA Connecticut Healthcare System, West HavenCT, USA; ^5^Department of Neuroscience, Yale University School of MedicineNew Haven, CT, USA; ^6^Interdepartmental Neuroscience Program, Yale University School of MedicineNew Haven, CT, USA

**Keywords:** individual difference, behavioral approach, behavioral inhibition, cognitive control, probabilistic learning

## Abstract

Proactive control allows us to maneuver a changing environment and individuals are distinct in how they anticipate and approach such changes. Here, we examined how individual differences in personality traits influence cerebral responses to conflict anticipation, a critical process of proactive control. We explored this issue in an fMRI study of the stop signal task, in which the probability of stop signal – p(Stop) – was computed trial by trial with a Bayesian model. Higher p(Stop) is associated with prolonged go trial reaction time, indicating conflict anticipation and proactive control of motor response. Regional brain activations to conflict anticipation were correlated to novelty seeking (NS), harm avoidance (HA), reward dependence, as assessed by the Tridimensional Personality Questionnaire, with age and gender as covariates, in a whole-brain linear regression. Results showed that increased anticipation of the stop signal is associated with activations in the bilateral inferior parietal lobules (IPL), right lateral orbitofrontal cortex (lOFC), middle frontal gyrus (MFG), anterior pre-supplementary motor area (pre-SMA), and bilateral thalamus, with men showing greater activation in the IPL than women. NS correlated negatively to activity in the anterior pre-SMA, right IPL, and MFG/lOFC, and HA correlated negatively to activity in the thalamus during conflict anticipation. In addition, the negative association between NS and MFG/lOFC activity was significant in men but not in women. Thus, NS and HA traits are associated with reduced mobilization of cognitive control circuits when enhanced behavioral control is necessary. The findings from this exploratory study characterize the influence of NS and HA on proactive control and provide preliminary evidence for gender differences in these associations.

## Introduction

As heritable personality traits (Elliot and Thrash, 2002, 2010), approach and avoidance motivation may exert a strong influence on cognition (Cloninger, 1987; Carver et al., 2000; Elliot and Thrash, 2002). A few studies have investigated the influences of approach and avoidance traits on the neural processes of proactive control. For example, in an AX-CPT with baseline, reward and penalty conditions, participants responded faster in the reward as compared to baseline condition (Locke and Braver, 2008). Sustained cue-related activity during reward in the right prefrontal cortex was positively correlated with behavioral approach sensitivity. In an emotional face Stroop task in which the proportion of incongruent trials varied between 35 and 65%, people high in trait anxiety demonstrated decreased activity in left ventrolateral prefrontal cortex, anterior insula, and OFC to incongruent trials when they expected a higher level of conflict (Krug and Carter, 2012). A risky or conservative mental set differentiates cerebral responses to errors under a reward contingency in the SST (Winkler et al., 2013). In an event-related potential study, individuals with higher threat-sensitivity displayed greater N2 to happy relative to fearful NoGo faces, suggesting that a mismatch between one’s temperament and the valence of the NoGo stimulus elevates the need for cognitive control (Pornpattananangkul et al., 2015). These studies suggest a relationship between approach/avoidance traits and cerebral responses during cognitive control in reward or emotion related contingencies, although the extant findings do not allow a clear conclusion as to how these personality traits modulate cerebral responses to support behavior. Further, it remains unclear whether or how the influence of approach/avoidance traits extends to cognitive motor control that does not explicitly implicate reward or affect processing.

There are several lines of thoughts in defining and operationalizing approach and avoidance in the personality literature, including Gray’s model of behavioral activation and inhibition systems (Carver and White, 1994), Eysenck’s (1997) theory of extraversion and neuroticsm and Cloninger et al.’s (1993) psychobiological model of novelty seeking (NS) and harm avoidance (HA). Although these personality theories differ in details, they all capature the fundamental psychological constructs of approach and avoidance (Hewig et al., 2005, 2006). In the present study, we operationalize approach and avoidance traits as NS and HA as defined by Cloninger et al. (1993). Research indicates that these dimensions are linked to the dopaminergic (approach) and serotonergic (avoidance) pathways (Hansenne and Ansseau, 1999; Schinka et al., 2002; Golimbet et al., 2007). NS describes a tendency to respond with intense excitement to novel stimuli, leading to pursuit of reward, whereas HA is defined as a tendency to respond to previously established aversive stimuli and to passively avoid punishment. These two dimensions are largely independent (Cloninger et al., 1993).

Personality traits exert a broad top-down influence on how people respond to contextual information (Fischer et al., 2015). People high in NS and HA show heightened attention each to novel, rewarding stimuli and to salient, aversive events. Such attentional bias may compromise their ability in utilizing context information for proactive control. Thus, we speculate that participants with higher NS and HA may demonstrate altered neural processes for proactive control. In addition, compared to women, men appear to show a higher score on sensation seeking (Lang et al., 2007) and a lower score on HA (Westlye et al., 2011). Gender differences also play an important role in cognitive control, including post-error slowing (Li et al., 2009) and interference inhibition in the Simon task (Christakou et al., 2009). Thus, gender differences should be taken into consideration in the examination of how approach/avoidance personality traits influence the neural correlates of proactive control.

In proactive control, we prepare for a changing environment on the basis of previous experience (Braver, 2012; Jahfari et al., 2012; Krug and Carter, 2012). Proactive control has been studied in the laboratory with a number of behavioral tasks including the Continuous Performance Task (CPT; Locke and Braver, 2008; Braver et al., 2009; Lesh et al., 2013), flankers task, Stroop task, set switching paradigms (Rushworth et al., 2001; Savine and Braver, 2010), and stop signal task (SST; Luks et al., 2007; Aarts et al., 2008; Jahfari et al., 2012; Krug and Carter, 2012). A cue is used to inform an upcoming conflict and the extent of conflict can be manipulated by changing the proportion of incongruent trials, where a change in behavioral responses is required.

In our recent study of the SST, a Bayesian approach was used to compute the probability of stop signal – p(Stop) – trial by trial based on the history of events. This estimate allowed us to delineate the neural correlates of conflict anticipation, a critical basis for proactive control (Harlé et al., 2014; Hu et al., 2015a,b). In behavior, higher p(Stop) was associated with prolonged go trial reaction time (GoRT) – a sequential effect – indicating proactive control of motor response (Hu et al., 2015a,b; Ide et al., 2015). That is, individuals slow down in response when they expect to encounter a stop signal. In fMRI, the anterior pre-supplementary motor area (pre-SMA) responds to higher p(Stop) or conflict anticipation. This finding replicated an earlier work of cued SST, where conflict anticipation recruited the right inferior frontal gyrus, pre-SMA, right inferior parietal lobule (IPL), and left insula (Jahfari et al., 2012).

Here, we built on our previous work (Ide et al., 2013; Hu et al., 2015a, 2016) and combined a Bayesian model with fast, event-related fMRI of the SST to investigate how NS and HA modulate the neural processes of proactive control. As discussed earlier, the Bayesian Dynamic Model allowed us to quantify the extent of conflict anticipation, as indexed by the trial-by-trial estimate of the likelihood of an impending stop signal. Further, by associating the extent of conflict anticipation to GoRT, we were able to characterize the behavioral consequence of conflict anticipation. We assessed a large cohort of healthy participants with Cloninger’s (1985, 1987) tridimensional personality questionnaire, examined how behavioral performance and regional brain activations during proactive control varied with NS and HA, and noted gender differences where relevant. Specifically, we explored the hypothesis that individuals with high NS and HA would each demonstrate diminished and increased response to conflict and post-conflict behavioral adjustment, respectively. Given that the hypothesis is very broad, the current study should be considered as exploratory.

## Materials and Methods

### Participants and Behavioral Task

A total of 78 healthy adults (48 females; age 30.2 ± 10.2 years; all right-handed) were recruited from the greater New Haven area through advertisements to participate in the study. All participants reported no current or history of major medical, neurological or psychiatric illnesses. None reported use of illicit substances and all tested negative in urine toxicology on the day of fMRI. All participants signed a written informed consent, in accordance to a protocol approved by the Yale Human Investigation Committee.

We employed a simple RT task in this stop signal paradigm (Hu et al., 2014, 2016). There were two types, “go” and “stop,” randomly intermixed in presentation with an inter-trial-interval of 2 s. A fixation dot appeared on the screen to engage attention at the beginning of a go trial. After a randomized time interval anywhere between 1 and 5 s (drawn from a uniform distribution), the dot turned into a circle, prompting participants to quickly press a button. The circle vanished at button press or after 1 s had elapsed, whichever came first, and the trial terminated. A premature button press prior to the appearance of the circle also terminated the trial. Approximately three quarters were go trials. The remaining one quarter were stop trials. In a stop trial, other than the fixation dot and go signal, an “X” (the stop signal) appeared after and replaced the go signal, instructing participants to withhold button press. Likewise, a trial terminated at button press or after 1 s if the participant successfully withheld the response. The time between the go and stop signals, the stop signal delay (SSD), started at 200 ms and varied from one stop trial to the next according to a staircase procedure, increasing and decreasing by 67 ms each after a successful and failed stop (Levitt, 1971). With the staircase procedure, we anticipated that participants would succeed in withholding the response half of the time. Participants were instructed to respond to the go signal quickly while keeping in mind that a stop signal could come up in a small number of trials, and both accuracy and response speed were emphasized (Li et al., 2008). Prior to the fMRI study, participants practiced on the same behavioral task outside the scanner. In the scanner, they completed four 10-min sessions of the task, with approximately 100 trials in each session.

On the basis of the race model (Logan, 1994), we computed for each participant the stop signal reaction time (SSRT), which represents the time one requires to stop the button press after the stop signal appears. Following our earlier work (Li et al., 2008), we estimated the critical SSD, the delay that allows a participant to correctly inhibit response to a stop signal in half of the stop trials, and computed the SSRT by subtracting the critical SSD from the median go trial RT.

### Trial by Trial Bayesian Estimation of the Likelihood of a Stop Signal

As in our previous work (Ide et al., 2013), we used a dynamic Bayesian model (Angela and Cohen, 2009) to estimate the prior belief of an impending stop signal on each trial, based on stimulus history. The model assumes that participants believe that stop signal frequency *r*_k_ on trial *k* has probability α of being the same as *r*_k-1_, and probability (1-α) of being re-sampled from a prior distribution π(*r*_k_). Participants are also assumed to believe that trial *k* has probability *r*_k_ of being a stop trial, and probability 1*-r*_k_ of being a go trial. Based on these generative assumptions, participants use Bayesian inference to update their prior belief of seeing a stop signal on trial *k*, *p*(*r*_k_|***s*_k_**_-_**_1_**) based on the prior on the last trial *p*(*r*_k-1_|***s*_k_**_-_**_1_**) and last trial’s true category (*s*_k_=1 for stop trial, *s*_k_=0 for go trial), where ***s*_k_** = {*s*_1_,…, *s*_k_} is short-hand for all trials 1 through *k*. Given that the posterior distribution was *p*(*r*_k-1_| ***s*_k_**_-_**_1_**) on trial *k*-*1*, the prior distribution of stop signal in trial *k* is given by:

$$p(r_{k}|s_{k-1})=\alpha p\left(r_{k-1}|S_{k-1}\right)+\left(1-\alpha \right)\pi (r_{k}),$$

where the prior distribution π(*r*_k_) is assumed to be a beta distribution with prior mean *pm*, and shape parameter *scale*, and the posterior distribution is computed from the prior distribution and the outcome according to the Bayes’ rule:

$$p(r_{k}|s_{k})\;\,\alpha \,\;P\left(S_{k}|r_{k}\right)p\left(S_{k-1}\right).\,\;\;\;\;\;\;\;\;\;\;\;\;\;\;\;\;\;\;\;\;\;\;\;\;\;\;\;\;\;\;\;\;\;\;\;\;\;\;\;\;\;\;\;\;\;\;\;\left(1\right)$$

The Bayesian estimate of the probability of trial *k* being stop trial, which we colloquially call p(Stop) in this paper, given the predictive distribution *p*(*r*_k_|***s*_k_**_-_**_1_**) is expressed by:

$$p\left(s_{k}=1|s_{k-1}\right)=\int \;P(s_{k}=1|r_{k})P\left(r_{k}|s_{k-1}\right){dr}_{k}=\int r_{k}\;P\left(r_{k}|s_{k-1}\right){dr}_{k}=(r_{k}|s_{k-1}).\;\;\;\;\;\;$$

In other words, the probability p(Stop) of a trial *k* being a stop trial is simply the mean of the predictive distribution *p*(*r*_k_|***s*_k_**_-_**_1_**). The assumption that the predictive distribution is a mixture of the previous posterior distributions and a generic prior distribution is essentially equivalent to using a causal, exponential, linear filter to estimate the current rate of stop trials (Angela and Cohen, 2009). In summary, for each subject, given a sequence of observed go/stop trials, and the three model parameters {α, *pm*, *scale*}, we estimated p(Stop) for each trial.

A sequential effect was quantified by Pearson correlation between p(Stop) – the Bayesian estimation of the probability of a stop signal – and RT on go trials for each participant (Ide et al., 2013; Hu et al., 2015a).

### Tridimensional Personality Questionnaire

All participants were assessed with the Cloninger’s Tridimensional Personality Questionnaire—Short Form (TPQ-short; Sher et al., 1995). Derived from the 100-item long form of the TPQ (Cloninger, 1987), the TPQ-Short demonstrated reliability and validity (Sher et al., 1995). It consists of 44 yes/no questions which cover the three dimensions: NS (13 items), HA (22 items), and reward dependence (RD; 9 items). Each personality subscale score was calculated by summing the item scores, reverse scored where necessary. A higher subscore each represents a higher level of NS, HA, and RD.

### Imaging Protocol

Conventional T1-weighted spin echo sagittal anatomical images were acquired for slice localization using a 3T scanner (Siemens Trio) with a 12 channel head coil. Anatomical images of the functional slice locations were next obtained with spin echo imaging in the axial plane parallel to the AC-PC line with TR = 300 ms, TE = 2.5 ms, bandwidth = 300 Hz/pixel, flip angle = 60°, field of view = 220 × 220 mm, matrix = 256 × 256, 32 slices with slice thickness = 4 mm, and no gap. Functional, blood oxygenation level dependent (BOLD) signals were then acquired with a single-shot gradient echo echo-planar imaging (EPI) sequence. Thirty-two axial slices parallel to the AC-PC line covering the whole brain were acquired with TR = 2000 ms, TE = 25 ms, bandwidth = 2004 Hz/pixel, flip angle = 85°, field of view = 220 × 220 mm, matrix = 64 × 64, 32 slices with slice thickness = 4 mm, and no gap. Slice scanning order was ascending interleaved. Three hundred images were acquired in each session for a total of four sessions.

### Data Analysis and Statistics

Data were analyzed with Statistical Parametric Mapping version 8 (SPM8, Wellcome Department of Imaging Neuroscience, University College London, UK). Images from the first five TRs at the beginning of each run were discarded to enable the signal to achieve steady-state equilibrium between RF pulsing and relaxation. Images of each individual participant were first corrected for slice timing and realigned (motion-corrected). A mean functional image volume was constructed for each participant for each run from the realigned image volumes. These mean images were normalized to an MNI (Montreal Neurological Institute) EPI template with affine registration followed by non-linear transformation (Friston et al., 1995; Ashburner and Friston, 1999). The normalization parameters determined for the mean functional volume were then applied to the corresponding functional image volumes for each participant. Finally, images were smoothed with a Gaussian kernel of 8 mm at Full Width at Half Maximum. The data were high-pass filtered (1/128 Hz cutoff) to remove low-frequency signal drifts.

Four main types of trial outcome were distinguished: go success (GS), go error (GE), stop success (SS), and stop error (SE) trial. In the general linear model (GLM), we modeled BOLD signals by convolving the onsets of the fixation point – the beginning of each trial – with a canonical hemodynamic response function (HRF) and the temporal derivative of the canonical HRF (Friston et al., 1994). Realignment parameters in all six dimensions were also entered in the model. We included the following variables as parametric modulators in the model: p(Stop) of GS trials, SSD of SS trials, p(Stop) of SS trials, SSD of SE trials, p(Stop) of SE trials, in that order. Inclusion of these variables as parametric modulators improves model fit (Buchel et al., 1996, 1998; Cohen, 1997; Hu et al., 2015a). The parametric modulator of p(Stop) would allow us to examine the neural correlates of stop signal or conflict anticipation. Serial autocorrelation of the time series was corrected by a first degree autoregressive or AR(1) model (Friston et al., 2000; Della-Maggiore et al., 2002). In the first analysis, we obtained for each participant contrasts “1” and “-1” on the parametric modulator “p(Stop)” on GS trials to examine how deviations from the average BOLD amplitude are modulated positively and negatively by trial-by-trial estimate of the likelihood of a stop signal. That is, in one-sample *t*-tests these two contrasts identified voxels with activation increasing (GS_p(Stop) > 0) and decreasing (GS_p(Stop) < 0) with the likelihood that a stop signal would appear.

In the second level analysis, results of one-sample *t*-tests were reported for clusters that survived peak voxel *p* < 0.05, corrected for family wise error (FWE) of multiple comparisons or a combination of voxel peak *p* < 0.001, uncorrected and cluster *p* < 0.05, FWE corrected.

In the whole brain regression, the *con* or contrast images of “GS_p(Stop) > 0” and “GS_p(Stop) < 0 in the first level analysis were used for the second level group statistics. These images were correlated with the NS, HA, and RD scores with age and gender as covariates in a simple regression across participants. As previous studies have demonstrated age and gender differences in the neural processes of cognitive control, we included these variables in the analyses (Li et al., 2006, 2009; Hu et al., 2012). Results were reported at *p* < 0.05, FWE corrected, using masks identified from the contrasts of “GS_p(Stop) > 0” and “GS_p(Stop) < 0, respectively. All voxel activations were presented in MNI coordinates.

## Results

### TPQ Measures and Stop Signal Task Performance

Across subjects, mean (±standard deviation) scores for NS, HA, and RD were 4.1 ± 2.5, 6.5 ± 4.4, and 6.4 ± 2.4, respectively. We tested for correlations between NS, HA, and RD, using an alpha of 0.05/3 = 0.0167 to guard against Type I error. NS, HA, and RD did not show any significant pair-wise correlation across subjects (NS/HA, *r* = 0.224, *p* = 0.049; NS/RD, *r* = -0.049, *p* = 0.671; HA/RD, *r* = -0.162, *p* = 0.157). We compared men and women and there was significant gender difference in HA (*t*_76_ = 2.372, *p* = 0.020; Cohen’s *d* = 0.556), with women showing higher HA score than men (7.44 ± 4.40 vs. 5.07 ± 4.12), but not in NS or RD (*t*_76_ = -0.403, *p* = 0.688; Cohen’s *d* = 0.095; *t*_76_ = -1.067, *p* = 0.289; Cohen’s *d* = 0.244, respectively).

In the SST, the average go response rate was 97.8 ± 3.2% (mean ± standard deviation) and the SS rate was 52.4 ± 3.4%. Average GoRT and SSRT were 634 ± 114 ms and 204 ± 42 ms, respectively. These measures are typical of SST performance and suggest that participants’ performance was well tracked by the staircase procedure. Examination of the sequential effect – the correlation between Go RT and p(Stop) – showed that 73 of the 78 participants demonstrated a significant sequential effect (*p* < 0.05) with an average sequential effect of 0.33 ± 0.13 (mean ± standard deviation). The latter finding suggested that conflict anticipation is associated with behavioral control.

There was no significant gender difference in SSRT (*t*_76_ = -1.825, *p* = 0.072; Cohen’s *d* = 0.428) or the sequential effect (*t*_76_ = 0.898, *p* = 0.372; Cohen’s *d* = 0.206). NS, HA and RD did not show a significant correlation with SSRT (*r* = 0.107, *p* = 0.351; *r* = -0.081, *p* = 0.481; *r* = -0.021, *p* = 0.856, respectively) or with the sequential effect (*r* = 0.012, *p* = 0.916; *r* = 0.062, *p* = 0.588; *r* = 0.026, *p* = 0.819, respectively).

### Conflict Anticipation: Regional Activations Modulated by p(Stop)

**FIGURE 1:**
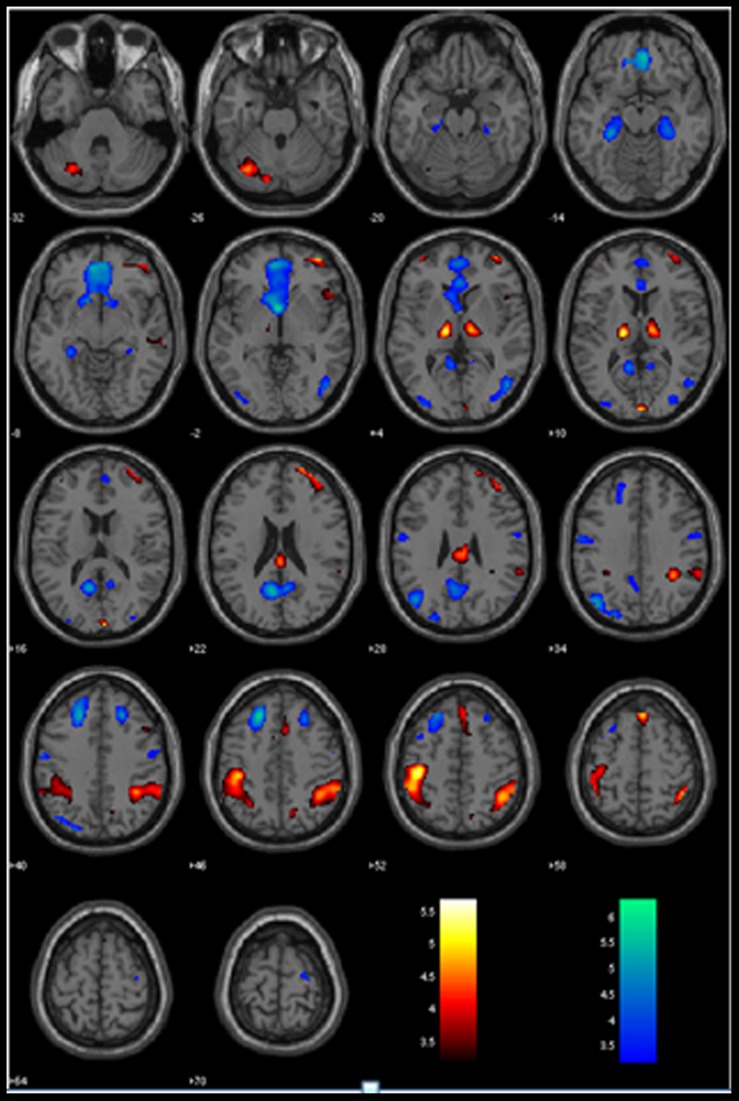
**Regional activations to stop signal anticipation.** Hot/winter color each shows activations to increased (GS_pStop > 0) and decreased (GS_pStop < 0) stop signal anticipation. BOLD contrasts were overlaid on a structural template in axial sections. Color bars indicate voxel *T* values.

The GLM showed that increased anticipation of the stop signal (GS_p(Stop) > 0) was associated with activations in the bilateral IPLs, right lateral orbitofrontal cortex (OFC) and middle frontal gyrus (MFG), anterior pre-SMA, and bilateral thalamus (**Figure 1**; **Table 1A**). Anticipation of the stop signal was also associated with less activations (GS_p(Stop) < 0) in the anterior cingulate cortex, bilateral superior frontal gyrus, posterior cingulate cortex, bilateral parahippocampal gyrus, and left angular gyrus (**Figure 1**; **Table 1B**). In addition, men showed greater activation in the left IPL (peak at *x* = -42, *y* = -43, *z* = 49) than women. There were no significant regional brain activations in association with age.

**Table 1:** Regional activations to (A) increased [GSp(Stop) > 0] and (B) decreased [GSp(Stop) < 0] stop signal anticipation.

Cluster size (#voxels)	Voxel *Z* value	MNI coordinate (mm)			Side	Identified brain region
		*x*	*y*	*z*		
**(A) Increased stop signal anticipation**						
88	5.18	-15	-16	10	L	Thalamus/Ventrolateral nucleus
540	5.04	-42	-25	52	L	IPL/Lateral fissure, post. Segment/SMG
	4.52	-48	-43	55		
	4.49	-48	-37	49		
236	4.92	24	59	25	R	Lateral OFC/MFG
	4.84	36	59	1		
	4.36	39	47	25		
96	4.66	12	-13	7	R	Thalamus/Ventrolateral nucleus
113	4.60	3	32	58	R	Anterior pre-SMA
	3.89	6	20	49		
	3.21	12	8	55		
485	4.58	45	-49	55	R	IPL/SMG
	4.44	57	-43	49		
	4.41	36	-46	37		
146	4.30	-33	-70	-26	L	Cerebellum
	4.11	-27	-70	-32		
	4.04	-12	-79	-26		
**(B) Decreased stop signal anticipation**						
845	5.08	6	41	-14	R	Medial OFC/rostral anterior cingulate cortex
	4.97	-3	50	-5	L	
287	5.35	-21	29	43	L	Superior frontal gyrus
	3.81	-39	11	55	L	
314	5.05	-9	-58	19	L	Posterior cingulate cortex
	4.48	-9	-52	10	L	
	4.35	9	-58	22	R	
103	4.79	-27	-34	-14	L	Parahippocampal gyrus
	4.06	-21	-22	-17	L	
199	4.74	-42	-76	34	L	IPL/Angular gyrus
	4.21	-24	-85	34	L	
83	4.68	21	32	40	R	Superior frontal gyrus
	3.69	30	32	52	R	
147	4.58	51	-70	4	R	Middle occipital gyrus
	3.66	33	-85	7	R	
94	4.42	27	-22	-17	R	Parahippocampal gyrus
	4.32	30	-31	-17	R	

*IPL, inferior parietal lobule; MFG, middle frontal gyrus; OFC, orbitofrontal cortex;SMA, supplementary motor area; SMG, supramarginal gyrus. All peak activations 8 mm apart are identified.*

### Regional Activations to Conflict Anticipation and the TPQ

**FIGURE 2:**
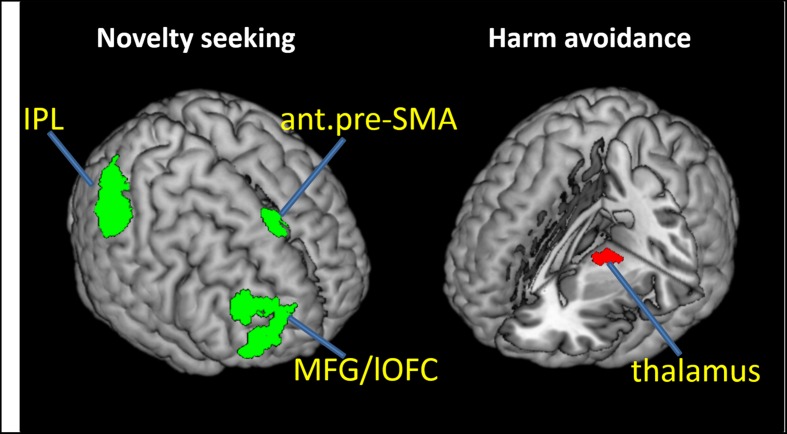
**Neural correlates of proactive control that negatively correlate with novelty seeking (NS; **Left)** and harm avoidance (HA; **Right)**.** ant.pre-SMA, anterior pre-supplementary motor area; MFG, middle frontal gyrus; lOFC, lateral orbitofrontal cortex; IPL, inferior parietal lobule. The IPL cluster is largely confined to the supramarginal gyrus.

We carried out a whole brain linear regression with the contrast of GS_p(Stop) > 0 as the dependent variable and each of the TPQ subscore (NS, HA, and RD), age, male, and female as independent variables. The results showed a significant negative correlation between NS and anterior pre-SMA (*x* = 6, *y* = 32, *z* = 58), MFG/lateral orbitofrontal cortex (MFG/lOFC, *x* = 45, *y* = 41, *z* = 28; *x* = 30, *y* = 62, *z* = 10) and the right IPL (*x* = 57, *y* = -40, *z* = 49) (**Figure 2** left panel; **Table 2A**). There was a significant negative correlation between HA and the thalamus (*x* = -9, *y* = -16, *z* = 1; *x* = -18, *y* = -16, *z* = 1; *x* = -18, *y* = -22, *z* = 4) (**Figure 2** right panel; **Table 2B**). There were no significant regional brain activations in association with RD.

**Table 2:** Regional activations to conflict anticipation in association with personality traits: Negative modulation of conflict anticipation (GS_p(Stop) > 0) by (A) Novelty seeking and (B) Harm avoidance.

Cluster size (# voxels)	Voxel *Z* value	MNI coordinate (mm)			Side	Identified brain region
		*x*	*y*	*z*		
**(A) Novelty seeking**						
28	2.60	6	32	58	L/R	Anterior pre-SMA
	2.56	6	26	58	L/R	
160	3.59	45	41	28	R	MFG/lOFC
	3.14	30	62	10	R	
347	3.25	57	-40	49	R	Inferior parietal lobule
**(B) Harm avoidance**						
69	2.73	-9	-16	1	L	Thalamus/Ventrolateral nucleus
	2.75	-18	-16	1	L	
	2.66	-18	-22	4	L	

*SMA, supplementary motor area, MFG, middle frontal gyrus; lOFC, lateral orbitofrontal cortex. All peak activations 8 mm apart are identified.*

**FIGURE 3:**
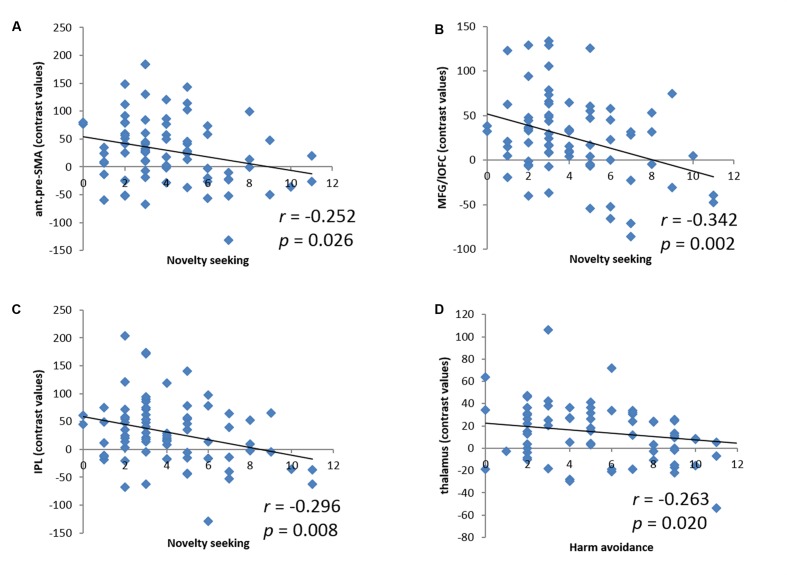
**Linear correlations between NS and the contrast values of **(A)** anterior pre-supplementary motor area (ant.pre-SMA), **(B)** middle frontal gyrus/lateral orbitofrontal cortex (MFG/lOFC), and **(C)** inferior parietal lobule (IPL); between HA and the contrast value of **(D)** thalamus.** Each dot represents one subject.

We derived the contrast values for all of these activity clusters for linear regressions. While these analyses did not provide any new information, they would help readers visualize the inter-subject variation. NS accounted for 6.3% (*r* = -0.252, *p* = 0.026, **Figure 3A**), 11.7% (*r* = -0.342, *p* = 0.002, **Figure 3B**), and 8.8% (*r* = -0.296, *p* = 0.008, **Figure 3C**) of the variance each for the contrast value of the anterior pre-SMA, MFG/lOFC, and IPL. In another linear regression HA accounted for 6.9% (*r* = -0.263, *p* = 0.020, **Figure 3D**) of the variance of the contrast value of the thalamus. Because of the skewed distribution of NS subscore, we performed a non-parametric, Spearman regression and showed that the correlations between NS and anterior pre-SMA, MFG/lOFC, and IPL largely remained significant (ρ = -0.218, *p* = 0.055; ρ = -0.278, *p* = 0.014; ρ = -0.246, *p* = 0.030, respectively).

We also carried out a whole brain multiple linear regression analysis with the contrast of GS_p(Stop) < 0 as the dependent variable and each of the TPQ subscore (NS, HA, and RD), age, male, and female as independent variables. The results showed no regional responses to any of the personality traits at the same statistical threshold.

### Gender Differences in the Relationship between Personality Traits and Cerebral Responses to p(Stop)

To examine gender differences, we compared the regression slopes between men and women for each correlation (Zar, 1999). For the clusters of anterior pre-SMA, IPL and thalamus, the slopes of linear regressions did not differ between men and women (*p*’s > 0.333). For the MFG/lOFC, the gender difference reached trend-level significance (*p* = 0.095). The negative association between NS and the MFG/lOFC activity in men (*r* = -0.538, *p* = 0.002) appeared to be stronger than that in women (*r* = -0.246, *p* = 0.092).

## Discussion

### Conflict Anticipation during the SST and Novelty Seeking (NS)

By estimating the probability of a stop signal or p(Stop) trial by trial with a dynamic Bayesian model, we reported that, with the likelihood of a stop signal increasing, participants proactively recruited the anterior pre-SMA, MFG/lOFC, bilateral IPL, bilateral thalamus, and cerebellum for proactive control. Further, the activation of anterior pre-SMA, MFG/lOFC, and IPL to conflict anticipation correlated negatively with the extent of NS, as assessed by the TPQ. Thus, higher NS is associated with less fronto-parietal activation to anticipated conflicts, broadly confirming our hypothesis.

Studies across methodologies including lesion (Picton et al., 2007), fMRI (Smittenaar et al., 2013; Zandbelt et al., 2013; Hu et al., 2015a), and transcranial magnetic stimulation (TMS; Chen et al., 2009; Soutschek et al., 2013) have suggested that the dorsomedial prefrontal cortex including the pre-SMA is critical for cognitive control. TMS of the pre-SMA selectively disrupted response selection (Soutschek et al., 2013). Independent component analysis showed that response inhibition engages a stopping network, including the pre-SMA, inferior frontal gyrus, and basal ganglia (Congdon et al., 2010; Zhang and Li, 2012). Consistent with the current results, people high in trait aggression (a personality trait positively related to NS) attenuated activation in pre-SMA and the motor cortex during response inhibition in an SST embedded in an emotional context.

Novelty seeking is also negatively associated with activation of the MFG/lOFC during conflict anticipation, consistent with its role in cognitive control (van Belle et al., 2014). In a Stroop task, adolescents low in sensation seeking demonstrated increased activation in inferior frontal gyrus extending to the OFC and frontal pole during proactive control (Andrews-Hanna et al., 2011). Contrasting No-go vs. go trials, Collins et al. (2012) observed greater activation in the MFG during response inhibition in the low sensation seekers, in accord with the current finding.

It is also worth considering these findings along with the work discussed earlier: Sustained cue-related right prefrontal cortical activity during a reward condition of the CPT was positively correlated with behavioral approach sensitivity (Locke and Braver, 2008). Individuals high in sensation seeking engaged the right MFG during Go response initiation in a Go/No-Go task (Collins et al., 2012). Thus, behavioral approach trait is associated each with greater and diminished right-hemispheric prefrontal activation during contingencies that encourage action and restraint of action, respectively. The negative association between NS and pre-SMA and MFG/lOFC activity is also consistent with previous results in occasional stimulant users, who showed weaker medial prefrontal activation during proactive control (Harlé et al., 2014). In addition, compared with healthy controls, recreational cocaine users displayed higher trait NS scores (Vonmoos et al., 2013), raising the possibility that NS and deficient proactive control may predispose individuals to stimulant misuse. Taken together, these results suggest a role of the pre-SMA and MFG/lOFC in anticipatory preparation and altered pre-SMA and MFG/lOFC activity during proactive control in novelty seekers. A propensity toward NS indicates a relative disregard for negative consequences in favor of the greater lure of positive consequence. People high in NS are likely to be less cautious, as reflected in decreased activation in the anterior pre-SMA and MFG/lOFC.

### Conflict Anticipation during the SST and Harm Avoidance (HA)

Thalamic activation to conflict anticipation negatively correlated with the tendency to avoid harm, an anxiety trait. According to the attentional control theory (Eysenck et al., 2007; Derakshan and Eysenck, 2009), anxiety increases the influence of stimulus-driven processing over goal-directed regulatory processes, resulting in poor attentional control which in turn impairs central executive functions such as inhibition.

During an antisaccade task, individuals with high trait anxiety showed lower frontocentral and central event-related potentials than those with low trait anxiety, in the preparatory period prior to target onset on correct antisaccade trials (Ansari and Derakshan, 2011). This finding suggests that anxiety interferes with efficient recruitment of top-down mechanisms required for the suppression of prepotent responses. Studies have implicated the thalamus in top down control (Dosenbach et al., 2008), such as task planning on the basis of external information in the Tower of London task (Wagner et al., 2006) and relocating attention during cued target detection (Hulme et al., 2010). In monkeys, neuronal activity in the ventroanterior and ventrolateral nuclei of the thalamus were enhanced during preparation for saccades compared with stimulus-driven prosaccades (Kunimatsu and Tanaka, 2010). In humans, the thalamus plays a key role in error-related cognitive control (Hendrick et al., 2010; Ide and Li, 2011). Using Granger causality analysis, Hwang et al. (2010) reported increased frontal thalamic connectivity during antisaccades compared to prosaccades. Thalamus increased activation following a cued than non-cued response in the CPT (Lütcke et al., 2009). Combining fMRI and electroencephalographic recording, Nagai et al. (2004) reported enhanced thalamus activity during contingent negative variation, an index of cortical arousal during action preparation and outcome anticipation. Together, a substantial body of evidence supports thalamic activity during proactive control. The top-down, task planning function, including conflict anticipation for proactive control, of the thalamus may thus be diminished in anxiety-prone individuals. On the other hand, the thalamus plays a central role in both cue-elicited gain and loss anticipation, encoding an “alerting” signal (Liu et al., 2011; Cho et al., 2013). Thus, an alternative explanation for the current finding is that, the thalamic alerting signal is less pronounced in people higher in HA.

An intriguing question is why HA is associated with decreased thalamic but not fronto-parietal activation during conflict anticipation. Considering again the attentional control theory (Eysenck et al., 2007; Derakshan and Eysenck, 2009), one is tempted to speculate that, in the SST, individuals with high trait HA recruit frontoparietal cortices for preparatory control but were unable to overide the influence of stimulus-driven processes in the thalamus. That is, anxiety biases the balance between a goal-directed and stimulus-driven attention system, resulting in decreased activation of the thalamus during proactive control. More research is warranted to investigate whether a full-blown imbalance in cortico-thalamic responses to proactive control of environmental stimuli may underlie anxiety disorders.

### Gender Differences

A direct contrast between genders revealed significantly higher left IPL activity in men as compared to women during proactive control. This finding is in line with earlier fMRI finding of greater parietal activation in men during post-error slowing (Li et al., 2009), working memory (Bell et al., 2006), visual-spatial selective attention (Rubia et al., 2010), and interference inhibition in the Simon task (Christakou et al., 2009). Thus, compared to women, men appeared to show greater reliance on a parietal mechanism for proactive control.

In addition, we found that the negative association between NS and the MFG/lOFC was significant only in men but not in women. As discussed above, decreased MFG/lOFC activation may reflect diminished stop preparation in the SST. The negative effect of NS on the MFG/lOFC activity during proactive control was significant in men but not in women, despite indistinguishable NS score, suggesting that men appear to be particularly vulnerable to the influence of NS trait on cognitive control.

Men and women are known to exhibit different clinical profiles in psychiatric conditions. Men are more frequently involved in externalizing disorders, which are known to implicate deficits in cognitive control (Luijten et al., 2013; Hu et al., 2015b; Ide et al., 2015), compared to women (Seedat et al., 2009). Previous imaging work has also demonstrated gender differences in the neural markers of externalizing including addiction disorders (Bednarski et al., 2012; Luo et al., 2013; Ide et al., 2014). The current findings thus provide additional insight into the sources of such differences and suggest a broader need for future studies to examine gender differences in systems and clinical neuroscience.

### Personality Traits and Cognitive Control May Share Underlying Neurobiological Mechanisms

Genetic evidence accumulates to suggest a potential mechanism that may contribute to the association between NS and HA and proactive control. Genetic variations in the catecholaminergic system contribute to individual differences in NS and HA, and modulate cerebral responses to cognitive control. Individuals carrying high-activity allelic variants of Monoamine Oxidase-A (MAO-A), a catabolic enzyme of monoamines, scored higher on NS (Shiraishi et al., 2006). High-activity carriers showed increased activity in the right ventrolateral prefrontal cortex activity and decreased activity in the right superior parietal cortex and bilateral extrastriate cortex during response inhibition in a Go/No-go task (Passamonti et al., 2006). Catechol O-methyltransferase (COMT) is another enzyme that catabolizes monoamines. Val and Met allele carriers of a COMT polymorphism are each associated with higher HA and NS (Kim et al., 2006; Golimbet et al., 2007). Importantly, COMT polymorphisms are known to modulate high-level cognitive processes, such as executive functions (Goldberg and Weinberger, 2004; Heinz and Smolka, 2006; Dickinson and Elvevag, 2009; Scheggia et al., 2012). In a modified Stroop task, where the predominance of incongruent and congruent contexts served to evoke proactive and reactive control, the anterior cingulate cortex and MFG each increased activity in Met and Val allele carriers during proactive control (Jaspar et al., 2014). These findings suggest a potential neurobiological link between NS and HA and proactive control.

### Limitations of the Study

Several limitations need to be considered. First, there were no correlations between personality traits and the magnitude of sequential effect. Thus, while NS and HA are associated with distinct neural processes for proactive control, these neural phenotypes do not translate to differences in behavioral performance. The seeming discrepancy between imaging and behavioral findings in relation to personality traits is not unique to the current study. For instance, activation of the nucleus accumbens during self-control differed between high and low impulsivity individuals despite a lack of differences in behavioral performance (Diekhof et al., 2012). Compared with stimulant-naïve control participants, occasional stimulant users demonstrated subtle alteration in inhibitory performance, but significantly attenuated neural activation related to proactive control in multiple brain areas (Harlé et al., 2014). It is possible that neural responses to proactive control are more sensitive to personality traits than behavioral measures. Second, there are other propositions on approach and inhibition traits in addition to Cloninger et al.’s (1993) model of NS and HA. Future work may consider Gray’s model of behavioral approach and behavioral inhibition (Luciana et al., 1992) systems (Carver and White, 1994), Eysenck’s (1997) theory of personality of extraversion and neuroticism to fully capture the influence of these personality dimensions on proactive control. Third, personality is known to have a robust genetic basis (e.g., Wang et al., 2014), as briefly discussed earlier. Future work incorporating genotyping will help evaluate whether neural phenotypes as revealed by fMRI are related to inter-subject variation in genetic predispositions and whether approach/inhibition traits and the neural processes of proactive control may share the same genetic bases. Finally, these findings are exploratory, as we did not formulate a specific hypothesis with respect to the influence of personality traits on proactive control or to the gender differences in such influences. Future work is needed to replicate and extend these findings.

## Conclusion

We reported how NS and HA personality traits influence the cerebral responses to conflict anticipation in the SST. Novelty seekers demonstrated decreased activity in the anterior pre-SMA, MFG/lOFC and IPL, and those who are more harm avoidant demonstrated decreased thalamic activation during conflict anticipation. The negative association between NS and MFG/lOFC activity appeared to be stronger in men than in women, a finding that requires replication.

## Ethical Standards

All procedures performed in studies involving human participants were in accordance with the ethical standards of the institutional research committee.

## Author Contributions

Conceived and designed the experiments: C-SL. Performed the experiments: SH, JM, and SZ. Analyzed the data: JH, SH, and SZ. Contributed to the writing of the manuscript: JH, SH, JM, HC, SZ, C-SL.

## Conflict of Interest Statement

The authors declare that the research was conducted in the absence of any commercial or financial relationships that could be construed as a potential conflict of interest.
